# Nitric oxide synthases, S-nitrosylation and cardiovascular health: From molecular mechanisms to therapeutic opportunities (Review)

**DOI:** 10.3892/mmr.2014.2968

**Published:** 2014-11-18

**Authors:** ADRIANA V. TREUER, DANIEL R. GONZALEZ

**Affiliations:** 1 Laboratory of Organic Synthesis, Institute of Chemistry of Natural Resources, University of Talca, Talca 3460000, Chile; 2 Department of Biomedical Basic Sciences, School of Health Sciences, University of Talca, Talca 3460000, Chile; 3 Interdisciplinary Excellence Research Program on Healthy Aging (Interdisciplinary Excellence Research Program on Healthy Aging), University of Talca, Talca 3460000, Chile

**Keywords:** S-nitrosylation, cardiovascular, nitric oxide synthase, heart

## Abstract

The understanding of nitric oxide (NO) signaling has grown substantially since the identification of endothelial derived relaxing factor (EDRF). NO has emerged as a ubiquitous signaling molecule involved in diverse physiological and pathological processes. Perhaps the most significant function, independent of EDRF, is that of NO signaling mediated locally in signaling modules rather than relying upon diffusion. In this context, NO modulates protein function via direct post-translational modification of cysteine residues. This review explores NO signaling and related reactive nitrogen species involved in the regulation of the cardiovascular system. A critical concept in the understanding of NO signaling is that of the nitroso-redox balance. Reactive nitrogen species bioactivity is fundamentally linked to the production of reactive oxygen species. This interaction occurs at the chemical, enzymatic and signaling effector levels. Furthermore, the nitroso-redox equilibrium is in a delicate balance, involving the cross-talk between NO and oxygen-derived species signaling systems, including NADPH oxidases and xanthine oxidase.

## 1. Introduction

Nitric oxide (NO) is a labile (half-life ~5 sec) and reactive free radical that is chemically able to diffuse within biological systems. The production of NO is catalyzed by a family of NO synthases (NOSs), which facilitate the nicotinamide adenine dinucleotide phosphate (NADPH)-dependent reaction of L-arginine with O_2,_ to yield NO and the amino acid L-citrulline. NOSs are homodimers and each subunit contains one flavin mononucleotide (FMN), one flavin adednine dinucleotide, one tetrahydrobiopterin and one Fe(3)-heme cofactor that facilitate the 5-electron oxidation of L-arginine to yield NO. The enzyme is activated by Ca^2+^ through its interaction with Ca^2+^-calmodulin.

Three isoforms of NOS have been sequenced and cloned: The neuronal isoform (NOS1 or NOS-1), the inducible isoform (iNOS or NOS2) and an endothelial isoform (NOS3), each encoded by different genes and on different chromosomes. The neuronal isoform was initially identified in neurons but later was found in a number of other tissues. Inducible NOS was first identified in activated macrophages, but its expression can be induced in several cell types through stimulation with cytokines or microbial products, such as lipopolysaccaride.

## 2. S-nitrosylation and redox mechanisms

Prototypic signaling was thought to occur via the activation of soluble guanylyl cyclase (sGC) leading to the production of cyclic guanosine monophosphate (cGMP). In the last 15 years, however, the importance of cGMP NO-dependent signaling has become increasingly clear, and this is now understood to be the primary mode of signaling. NO directly modifies the behavior of a diverse range of proteins by S-nitrosylation of cysteine residues (1,2). These reactions have been shown to be highly specific, highly regulated and to require substantially lower concentrations of NO compared with the generation of cGMP. To achieve specificity of protein modification, there is growing evidence that NOSs are often found as part of protein signaling complexes, in which S-nitrosothiol (SNO) signaling occurs (3,4). These provide local, and thus targeted, sources of NO.

S-nitrosylation involves the formation of a covalent bond between a nitrosomium equivalent (NO^+^), donated from N_2_O_3_ and a thiol (R-SH).

$${\text{N}}_{2}{\text{O}}_{3}+\text{R}-\text{SH}\to \text{R}-\text{SNO}+{\text{H}}^{+}+{{\text{NO}}_{2}}^{-}$$

Although the exact mechanism of this reaction is not fully resolved, certain mechanisms that may be involved in the formation of N_2_O_3_, a key intermediate in the nitrosylation reaction, have been proposed (3,4). NO reacts rapidly with the superoxide anion O2^•−^-(k~3.7×10^7^ M/s) to produce peroxynitrite ONOO^−^, which in turn reacts consecutively with a proton and another NO molecule to generate dinitrogen trioxide.

$$\begin{array}{l} \text{NO}+{{\text{O}}_{2}}^{•-}\to {\text{ONOO}}^{-}\\ {\text{ONOO}}^{-}+{\text{H}}^{+}\to \text{trans}-\text{ONOOH}\\ \text{trans}-\text{ONOOH}+\text{NO}\to {\text{NO}}_{2}+{{\text{NO}}_{2}}^{-}\\ {\text{NO}}_{2}+\text{NO}\to {\text{N}}_{2}{\text{O}}_{3} \end{array}$$

The dinitrogen trioxide generated is able to nitrosylate cysteine residues of proteins. It has also been shown that peroxynitrite is able to nitrosylate reduced glutathione (GSH) to generate S-nitrosoglutathione (GSNO)(5). However, the balance of NO and superoxide must be adequate, otherwise the reaction shifts towards a more oxidant chemistry (6,7). From this, it may be inferred that the nitrosylation of thiols depends critically on the redox environment from which NO and superoxide originate.

### Trasnitrosation

Transnitrosation is the process in which an NO^+^ equivalent is transferred from one molecule to another (8). The transfer among thiols is highly favored in comparison to other nitrogen or carbonyl groups.

$${\text{R}}^{\prime }-\text{SNO}+\text{R}-\text{SNO}\to \text{R}-\text{SNO}+{\text{R}}^{\prime }-\text{SH}$$

In cells and the interstitial space there is equilibrium between low molecular weight SNO and S-nitrosylated proteins. Within the cells, glutathione and L-cysteine are an important source of thiols groups, which are able to accept and transfer NO by transnitrosation (9).

### Catabolism of S-nitrosothiols

GSNO is one of the most abundant intracellular nitrosothiols and may act as an NO donor. Liu *et al* (10,11) described an enzyme, nitrosoglutathione reductase, which was able to catabolize GSNO. This enzyme was formerly termed a formaldehyde dehydrogenase. This study (10) showed, this enzyme metabolizes S-nitrosoglutathione to oxidized glutathione and ammonia, according to the following steps:

$$\begin{array}{l} \text{GSNO}+\text{NADH}+{\text{H}}^{+}\to \text{GSNHOH}+{\text{NAD}}^{+}\\ \text{GSNHOH}+\text{NADH}+{\text{H}}^{+}\to {\text{GSNH}}_{2}+{\text{NAD}}^{+}+{\text{H}}_{2}\text{O}\\ {\text{GSNH}}_{2}+\text{GSH}\to \text{GSSG}+{\text{NH}}_{3} \end{array}$$

This process is well-conserved throughout evolution, and has been identified in yeast, bacteria, plants and mammals. The activity of GSNO has been characterized as an important defense mechanism against nitrosative stress (an excess of NO output, primarily due to an increase in NOS2 activity). Its role in the cardiovascular system is under active investigation.

### Reaction with hemoglobin (Hb)

Hb binds O_2_ and NO. O_2_ is carried at hemes, and NO at hemes (Fe) and cysteine thiols. The O_2_/NO binding functions of Hb are principally governed by equilibrium between deoxy (T) and oxy (R) structures, according to the pO_2_. An allosteric change from R to T quaternary structure lowers the affinity of hemes for O_2_ and promotes the transfer of NO groups from SNO-Hb to acceptor thiols. In this way red blood cells may provide NO vasodilator activity in regions of low pO_2_, such as the peripheral microvasculature and the pulmonary circulation (12,13).

NO rapidly diffuses across cell membranes, although its reactivity prevents it from moving >1 mm from the membrane. In particular, it is extremely reactive with oxyhemoglobin and deoxyhemoglobin.

$$\text{NO}+{\text{HbO}}_{2}\to {{\text{NO}}_{3}}^{-}+\text{Hb}$$

### Activation of guanylate cyclase (GC)

The best characterized pathway for the biological effects of NO is the interaction with sGC. GC possesses a heme group, which reacts with NO (in its free radical form but not as a charged species), forming a penta-coordinated structure with the Fe^2+^ of the heme moiety and a histidine residue of one of the subunits of sGC. The exact mechanism whereby the binding of NO leads to the activation of this enzyme has not been yet been identified. However, it has been hypothesized that the formation of the penta-coordinated complex produces a conformational change that leaves the Fe^2+^ out of the configuration plane of the porfirinic ring. This change allows GTP to bind and thereafter be converted to cGMP (14,15). The concentration of NO that is able to activate sGC has been estimated at an EC_50_ ranging from 80–250 nM, depending on whether the experimental model used was a cell-free system, cells in culture or intact tissues (14,15). The regulation of intracellular cGMP levels is determined by the rate of it synthesis, but also by its degradation, which is catalyzed by phosphodiesterases. These are enzymes that convert cGMP to GMP. Of these, phosphodiesterase V is highly specific for cGMP degradation (16). Among the intracellular targets of cGMP are protein kinase G (PKG) and phosphodiesterase II (which cGMP modulates positively, leading to an increase in their activity), phosphodiesterase III (which it modulates negatively) and cyclic nucleotide-gated ion channels (17).

## 3. Biology of NO and nitric oxide synthases

**Figure 1:**
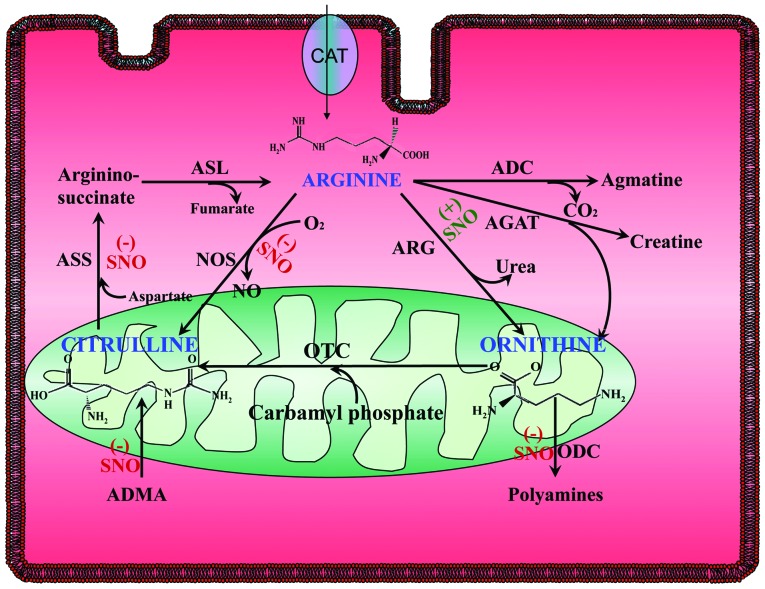
Regulation by SNO of NO-L-Arginine metabolism in the cell. SNO regulates positively (+) or negatively (−) a number of enzymatic steps involved in NO production via the metabolism of L-arginine. SNO, S-nitrosylation; NO, nitric oxide; ASS, argininosuccinate synthase; ADC, arginine decarboxylase; NOS, nitric oxide synthase; ARG, arginase; ASL, argininosuccinate lyase; DDAH, dimethylarginine dimethyl aminohydrolase; ODC, ornithine decarboxilase; AGAT, arginine:glycine amidinotransferase; OTC, ornithine transcarbamylase; CAT, cationic amino acids transporter.

Intracellularly, NO is synthesized from the amino acid L-arginine and O_2_. In turn, L-arginine is synthesized in the urea cycle, but may also enter cells from the plasma. In endothelial cells, L-arginine is incorporated by a system of transporters for cationic amino acids (CATS) (18,19). In the human myocardium CAT1 and CAT2b have been identified, whereas in the vasculature the subtypes present are CAT1 and CAT2a. Once inside the cell, L-arginine is converted to L-citrulline and NO by NOSs. L citrulline then enters the urea cycle to be recycled to L-arginine by argininosuccinate synthase and argininosuccinate lyase. Concomitantly, NOS competes for L-arginine with arginase. In endothelial cells, the majority of these enzymes are located in the caveolae (20). It is hypothesized that inside the cells, different pools of arginine and NOS exist. This may in part explain the ‘arginine paradox’. This is that in certain cell types, such as endothelial cells, even with significant intracellular concentrations of L-arginine, extracellular L-arginine influx through CATS is nevertheless required for NOS activity. By contrast, in cardiac myocytes, NOS1 located in the sarcoplasmic reticulum, is unaffected by extracellular arginine (21). Notably, L-arginine transport is compromised in a number of human diseases, including congestive heart failure (22) and gestational diabetes (23). Arginase hydrolyzes L-arginine to ornithine and urea, as part of the urea cycle in the mitochondria. It is thus able to modulate L-arginine bioavailability (21,24,25). Two types of mammalian arginase have been described, arginase I and II, which are encoded by different genes. Arginase I is located in the cytoplasm and is expressed most abundantly in the liver, whereas arginase II is a mitochondrial enzyme and is expressed primarily in extrahepatic tissue. Arginase I and II are expressed in mouse, cat and human myocardium whereas in endothelial cells arginase I is predominant. It has been shown to be upregulated in coronary arterioles in states of hypertension and by H_2_O_2_. In cardiac myocytes, arginase II interacts with and reciprocally regulates NOS1 (21). Recently, in cat myocytes a current of L-arginine that is probably due to the presence of a CAT2A transporter has been demonstrated. This current is coupled to NO production (26), and this is most likely to occur via the activity of NOS3, due to its caveolar localization, but this aspect remains to be determined. This highlights the importance of the subcellular localization of NOS3 in terms of its activation. Among the enzymes involved in arginine metabolism (Fig. 1), argininosuccinate synthetase (27), dimethylarginine dimethyl aminohydrolase (28,29) and ornithine decarboxilase (30), as well as NOS, are also reversibly inhibited by S-nitrosylation.

### Subcellular localization and trafficking of NOS

Initially, NOS3 was thought to be a strictly membrane-bound protein, localized to the caveolae. It later became evident that NOS3 is located in various intracellular compartments, depending on physiological status and pharmacological stimulation (24,31,32).

Due to myristate and palmitate acylation of NOS3, it is able to locate caveolae. These are discrete domains in the plasma membrane, rich in cholesterol and sphingolipids, which appear under electron microscopy as characteristic ‘flask-shaped’ invaginations. This organelle acts as a specialized site for signal transduction and as a mechanical sensor. Caveolae are abundant in endothelial cells and myocytes, where they express caveolin 1 and 3, respectively. Caveolin inhibits NOS3 activity, although this confinement is necessary for NOS3 activation, for both mechanical and agonist-induced stimulation. Agonists of NOS3 include neurotransmitters, such as adenosine triphosphate (ATP), acetylcholine and histamine; growth factors, such as vascular endothelial growth factor (VEGF); lipids, including platelet-activating factor and sphingosine phosphate 1; and kinins.

Caveolae are involved in mechano-sensing in endothelial cells and cardiac myocytes. Although primarily located in caveolae, NOS3 is able to translocate to alternative subcellular compartments, including the golgi apparatus, the cytosol and junctions between endothelial cells. NOS interacts with other proteins, including platelet endothelial cell adhesion molecule, dynamin, NOS traffic inducer (NOSTRIM) (33), and NOS-interacting protein (NOSIP) (34,35,36). Within caveolae, NOS3 is bound to caveolin-1, the main scaffolding protein of this organelle, and to dynamin-2, a large GTPase. NOS3-induced nitrosylation activates dynamin, and these proteins translocate to intracellular sites, tethered by NOSTRIN and NOSIP, which have been recently identified by use of the yeast two-hybrid system (33,34,35). As with the majority of enzymes involved in NO biology, NOS3 is nitrosylated, and the degree of nitrosylation varies depending on the subcellular compartment. Membrane bound NOS3 displays a higher degree of nitrosylation than NOS3 in the cytosol. In addition, agonist stimulation induces denitrosylation of NOS3 in a time-dependent manner (37,38). The enzymatic mechanisms underlying de-nitrosylation remain unknown.

In atrial and ventricular myocardium it has been shown that NOS3 can translocate from the plasma membrane to the cytosol upon β3 adrenergic stimulation (39,40). Classically, NOS3 activity has been associated with cGMP production (discussed later). Recently, growing evidence has shown that NOS3 is also able to induce S-nitrosylation in endothelial cells, and that this process is limited by distance (41), highlighting the importance of subcellular location. For instance, in vascular endothelial cells, two agonists that induce NOS3 activity, acetylcholine (Ach) and platelet-activating factor (PAF), cause the enzyme to become localized in different compartments. These agonists thereby produce different physiological responses. Ach induces vasorelaxation (30), whilst PAF induces vasoconstriction and increases vessel wall permeability (31). NOS3 is known to induce S-nitrosylation of N-ethylmaleimide-sensitive factor (NSF) (42), dynamin, thioredoxin (43), NOS3 itself (37,38,44) and arginine succinate (27) although this list is expected to increase.

### Subcellular localization and protein-protein interactions of NOS1

Within the cardiovascular system, NOS1 is primarily expressed in the heart and smooth muscle (45). In heart and skeletal muscle, the specific splice variant expressed is NOS1μ (46). This variant contains a unique 102-base pair (34 amino acid) insert between the calmodulin and FMN binding domains. Compared with NOS3, NOS1 lacks acylation sites but has a C-terminal PDZ domain, which facilitates unique protein-protein interactions. This domain anchors NOS1 and the α1-syntrophin-dystroglycan complex to the sarcolemma in skeletal muscle. This interaction is disrupted in several muscular dystrophies, which will be discussed later in the review. Through its PDZ domain, NOS1 also associates with the adaptor protein CAPON (C-terminal-PDZ of NOS1) at the cytosolic side, and to the plasma membrane Ca ATPase (47), which negatively regulates the activity of NOS1 in the heart (48,49). CAPON competes with PSD95/93 for binding to NOS1 and may be involved in the mechanisms that govern NOS1 translocation. In the heart, the plasmalemmal Ca^2+^ pump, PMCA4b and α-syntrophin form a tertiary complex with NOS1. Within this complex syntrophin and PMCA negatively modulate NOS1 activity. Furthermore, in skeletal muscle NOS1 binds to protein inhibitor of NOS (PIN), although the role of this small protein in NOS1 activity is unclear. In the sarcoplasmic reticulum (SR) of cardiac myocytes, NOS1 may also bind to the ryanodine receptor (RyR) 2 (50) and xanthine oxidoreductase (XO) (51). Thus it appears that at least two pools of NOS1 may exist in the heart. One that is localized to the sarcolemma (as in skeletal muscle) and another to the sarcoplasmic reticulum. At these sites, these pools may become closely apposed and functionally difficult to distinguish. This hypothesis requires further investigation.

## 4. NO in heart failure: Nitroso-redox disequilibrium

NOS2 is not usually expressed in the healthy heart, but it may be induced by pro-inflammatory cytokines and endotoxins, such as lipopolysaccaride, and in heart failure (HF). Interleukin-6 (IL-6) induces *de novo* synthesis of NOS2 through the Janus kinase 2/signal transducer and activator of transcription 3 pathway. Since NOS2 activity is independent of increases in [Ca^2+^]_i_, it is hypothesized to produce NO in an unregulated manner. This enzyme has previously been detected in models of septic shock. In this condition, NOS2 is able to reduce contractility by decreasing intra-SR Ca^2+^ content following exposure of cardiomyocytes to LPS. This effect appears to be mediated by decreased phospholamban (PLN) phosphorylation. In other models, induction of NOS has been shown to decrease contractility. In myocytes from patients with HF, NOS2 is known to be upregulated, and its pharmacological inhibition restores the β-adrenergic response (52). In this situation, the process that is affected also appears to be Ca^2+^ cycling. Recently, it has been shown that septic shock leads to nitroso-redox disequilibrium; where increased NO production from NOS2 and superoxide from XO lead to profound disturbances in calcium cycling, in particular causing diastolic leakage (53), a process that is likely to be mediated by oxidation of cysteine residues of RyR (54) by reactive oxygen species (ROS) or peroxynitrite. As discussed later, NOS2 may also be important in the pathogenesis of viral myocarditis.

### Role of NO in cardiomyopathy associated with Duchenne, Becker and X-linked muscular dystrophy

By virtue of its PDZ domain, unique among NOS isoforms, NOS1 is able to bind α1-syntrophin, a cytoskeletal protein and a member of the dystrophin protein complex, which is involved in organization at the cytoskeleton-plasma membrane interface. Loss of dystrophin and the subsequent disruption of the dystrophin complex from the sarcolemma ultimately lead to muscle degeneration in patients with X-linked, Duchenne and Becker muscular dystrophy, X-linked conditions caused by mutations in the dystrophin gene. Disruption of the dystrophin complex has also been implicated in acquired forms of dilated cardiomyopathy and in cardiomyopathy occurring as a result of viral infection. Loss of dystrophin also leads to the loss of NOS1 in cardiac and skeletal muscle (55). This ‘natural’ NOS1 deficiency is observed in mdx mice (a mouse model of Duchenne muscular dystrophy), which is associated with dysfunction and altered Ca^2+^ cycling of the heart, as well as significant electrocardiogram abnormalities, including deep Q waves and polyphasic R waves.

As cardiomyopathy progresses, arrhythmias and conduction abnormalities may occur (56). Whilst the increased fibrosis observed in the hearts of patients with this condition may responsible for this dysfunction, the changes in Ca^2+^ regulation due to the loss of NOS1 may also be important in this process. For example, cardiac myocytes in mdx mice display signs of Ca^2+^ leakage, such as increased diastolic [Ca^2+^]_i_ (57and decreased post-rest potentiation (58). It is possible that loss of NOS1 in these dystrophinopthies increases the level of oxidative stress (59), and that this is associated with deregulated Ca^2+^ handling. A recent breakthrough in this field has shown that this phenotype can be rescued by inducing NOS1 over-expression in mdx mice (60).

### Viral cardiomyopathy

Enteroviral infections may induce myocarditis. The coxsackievirus B3 (CVB3), an enterovirus, has been associated with myocarditis in humans, and has been shown to induce myocarditis in a murine model. Following infection of the cardiomyocytes, the viral protease 2A degrades dystrophin at its 3 hinge region. As in Duchenne, X-linked and Becker muscular dystrophy, dystrophin degradation leads to cardiomyopathy. NO donors are able to nitrosylate a serine residue in the active site of the viral protease, inhibiting its activity (61). In animals models of coxsackievirus B3 infection, this nitrosylation is likely to be derived from NOS2, which is predominantly found in activated macrophages, since NOS2 knock-out mice displayed higher titers of viral infection (62,63). However, this does not exclude the possibility that it is NO derived from NOS1 or NOS3 that nitrosylates the protease and prevents dystrophin degradation. In an animal model, this inhibition can also be achieved through administration of NO donors, including synaptosomal-associated protein (SNAP) and organic nitrates (GTN and isosorbide dinitrate) (64). This mechanism has been recently investigated in a patient with enterovirus-induced myocarditis and cardiomyopathy (61).

## 5. Role of NO in the circulation

### Vascular tone

**Figure 2:**
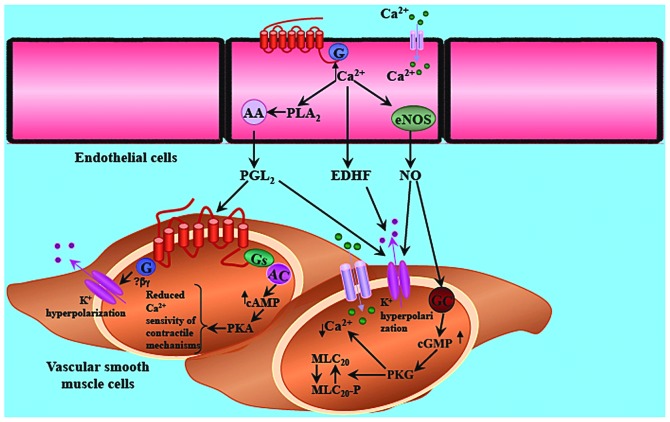
Regulation of vascular tone by NOS. The classical paradigm of vascular relaxation with NOS3-derived NO from endothelial cells diffusing into the adjacent vascular smooth muscle cells, where NO activates sGC. This increases the intracellular levels of cGMP, which in turns activates cGMP-dependent PKG. This kinase induces a series of phosphorylation that ultimately leads to a decrease in the degree in contraction via at least two mechanisms: Reduction of Ca^2+^ concentration and reduction in Ca^2+^ sensitivity. A reduction of Ca^2+^ concentration can be achieved by inhibiting Ca^2+^ influx through Ca^2+^-activated K^+^ channels. The cGMP pathway has been shown to activate this channel, which hyperpolarizes the layer of smooth muscle cells and indirectly inhibits the influx of Ca^2+^ through voltage-activated Ca^2+^ channels. In addition, the cGMP pathway also directly inhibits the voltage-activated Ca^2+^ channels. This inhibition can also be produced by direct S-nitrosylation of the channel. NOS, nitric oxide synthase; NO, nitric oxide; PLA_2_, phospholipase A_2_; MLC, myosin light chain; EDHF, endothelium derived hyperpolarizing factor; GC, guanylate cyclase; AC, adenylate cyclase; PKG, protein kinase G.

The role of NO in the regulation of vascular tone was one the first features attributed to this molecule in the seminal observations of Furchgott (65). The classical paradigm of vascular relaxation holds that the NOS3-derived NO from endothelial cells diffuses into adjacent vascular smooth muscle cells. There, NO activates sGC, increasing the intracellular levels of cGMP, which in turns activates cGMP-dependent PKG. This kinase induces a series of phosphorylation that ultimately results in a decrease in the degree of contraction via at least two mechanisms: A reduction of the Ca^2+^ concentration and a reduction in Ca^2+^ sensitivity (Fig. 2).

The reduction in Ca^2+^ concentration may be achieved by inhibiting the Ca^2+^ influx through Ca^2+^-activated K^+^ channels. The cGMP pathway has been shown to activate these channels, which hyperpolarize the layer of smooth muscle cells and indirectly inhibit the influx of Ca^2+^ through voltage-activated Ca^2+^ channels. In addition, the cGMP pathway can directly inhibit the voltage-activated Ca^2+^ channels. This inhibition is also achieved by direct S-nitrosylation of the channel (66). Furthermore, the [Ca^2+^]_i_ in smooth muscle can be reduced by activation of the plasma membrane Ca^2+^/ATPase pump (PMCA), which extrudes Ca^2+^ from the cell, and by activation of the Ca^2+^/ATPase pump in the sarcoplasmic reticulum (SERCA). NO is able to increase SERCA activity by S-glutathiolation of cysteine residues in the pump (67). The IP3 pathway, which is important in the Ca2+ signaling of a number of cell types, is inhibited by PKG phosphorylation of the IP3 receptor and by decreasing the generation of IP_3_ (68). Although vasorelaxation largely relies upon the NO-cGMP-PKG axis, nitrosothiols have been shown to induce a long-lasting relaxing effect, independently of PKG (69,70). This suggests that voltage-gated Ca^2+^ channels may be inhibited by S-nitrosylation (66), although this has not been addressed systematically thus far. Indeed, the role of NO in the maintenance of normal vascular tone has been highlighted by the fact that mice with a genetic deletion of NOS3 (but not NOS1 or NOS2 deficient mice) were hypertensive (71), an effect that is also observed with pharmacological inhibition of NOS. Recently, in healthy humans, the contribution of NOS to systemic blood pressure was estimated at ≥30 mmHg, during autonomic blockade (72).

### Endothelial dysfunction and nitroso-redox imbalance

Endothelial dysfunction may be attributable to a nitroso-redox imbalance. This is a syndrome characterized by decreased endothelium-dependent vasorelaxation. Aside from this impairment of vasomotion, other features of endothelial function are affected, including coagulation and the inflammatory response. This condition is observed in patients with hypertension, diabetes, atherosclerosis and heart failure (73). Decreased NO bioavailability appears to be central to the pathogenesis of this condition. Oxidative stress makes an important contribution to endothelial dysfunction. O2^•−^derived from NADPH oxidase and XO is important in this phenomenon, as it reduces the bioavailability of NO and probably generates peroxynitrite (74). In addition, uncoupled NOS is a potential source of O2^•−^ in the absence of appropriate cofactors, in particular tetrahydrobiopterin (75). Endothelial cells exposed to oscillatory shear stress increase their O2^•−^ production, which is derived in part from XO activity (76). Notably, XO inhibition with allopurinol has been shown to improve endothelial function in patients with heart failure, hypertension and diabetes (74,76,77). Furthermore, NO bioavailability is decreased by increased arginase activity, as arginase competes with NOS for L-arginine. In the cardiovascular system, two isoforms of arginase have been described in endothelial cells and cardiac myocytes (21,25,78,79).

It has been shown in models of older animals, that impaired endothelium-dependent vasorelaxation is restored by pharmacological inhibition or molecular ablation of arginase I (24). In addition, arginase inhibition has been shown to restore endothelial function in a rat model of hypertension (80). Arginase activity is enhanced by pro-atherogenic and pro-inflammatory agents, such as oxidized low density lipoprotein (81) and thrombin (82).

### Steady, oscillatory shear stress, NO and superoxide

Shear stress is the tangential force generated by blood flow against the walls of vessels. This flow is steady if the vessel is compliant. When blood vessels become stiff (as occurs in certain diseases or with aging), the blood flow becomes turbulent and oscillatory. Indeed, in the coronary circulation, the flow is oscillatory. Steady shear stress is a potent stimulus for the release of NO from endothelial cells, the inner cell lining of blood vessels. By contrast, oscillatory shear stress is a stimulus for the release of superoxide from NADPH oxidase and XO (76). Oscillatory flow, with the accompanying disturbed nitroso-redox state, generates an environment prone to the development of atherogenic lesions. The mechanism that links the oscillatory flow with the activation of oxidases is not yet known.

Steady shear stress induces the activity of NOS3, mainly through Akt-dependent phosphorylation of a serine residue (1177) (83). Endothelial cells are able to sense mechanical stimulation, although the exact mechanisms by which this signal is transduced have not been fully elucidated. It has been shown that an increase in shear stress activates the VEGF receptor 2 in a manner independent of ligand binding. In turn, VEGF receptor 2 phosphorylation activates the phosphorylation of Src. This protein phosphorylates the scaffolding protein Gab1 that, once activated, associates with the p85 subunit of PI3 kinase, activating Akt (84,85).

### Pre-eclammpsia and nitroso-redox imbalance

Pre-eclampsia is a common and potentially dangerous complication of pregnancy, particularly in developing countries. Pre-eclampsia is characterized by hypertension, proteinuria and edema (86). Genetic, immunological and environmental factors may all be involved in its etiology. However, the triggering to pre-eclampsia is a reduction in placental perfusion in the early stages of pregnancy (87). This hypoxic state leads to disturbance in the placental vasculature, particularly in the endothelial layer. Pre-eclampsia is a syndrome that in certain aspects is closely associated with endothelial dysfunction (88). The placental vasculature display abnormal reactivity, and reduced NO bioavailability appears to be central to this phenomenon (89). In the placental circulation, the role of NO in the regulation of vasomotor tone is more important than in other organs, since its lacks autonomic innervations. The placenta is known to be a site of NO production (90). Patients with pre-eclampsia display endothelial dysfunction, reduced plasma nitrite levels and increased levels of asymmetric dymethylarginine (88,89). However, increased levels of oxidative stress have been found in the placental vasculature of pre-eclamptic patients (91). In pre-eclampsia, the placental flow is reduced, which leads to hypoxia (92), a potent stimulus for the activation of XO. XO is expressed in cytotrophoblasts, syncythiotrophoblasts and stromal villious cells (93,93,94), but its role in the oxidative burst of pre-eclampsia has not been determined. Many *et al* (95) showed that in placental tissue of pre-eclamptic patients there is increased XO activity, associated with increased serum levels of uric acid. Although the precise role of XO in the pathogenesis of pre-eclampsia remains elusive, the prospect of antioxidant therapy has become increasingly attractive (96).

## 6. Inflammation

The inflammatory response consists of a number of sequential events. In the vasculature, endothelial-derived NO is known to regulate at least two key processes: Leukocyte and platelet attachment to the endothelial wall and the increase in vascular permeability that leads to leukocyte and solute infiltration. The post-capillary venules are the primary site for leukocyte (specifically, neutrophil) adhesion and transmigration, which are negatively regulated by NO (97). NO inhibits exocytosis of Weibel-Palade bodies, which contain the adhesion molecule, P-selectin, that is exposed on the cell surface to facilitate platelet attachment (98). At the molecular level, Matsushita et al *et al* (42) demonstrated that S-nitrosylation of N-ethylmaleimide sensitive fusion protein by NO results in destabilization of the exocytic machinery that tethers exocytic granules to fuse with the plasma membrane to expose the content of Weibel-Palade bodies to the surface. In the physiological context, the NOS3-derived NO from endothelial cells mediates this nitrosylation. In this way, NO exerts a basal anti-inflammatory effect on the attachment of platelets and leukocytes to the endothelium. Notably, when there is an inflammatory stimulus, NO is required to increase the permeability of the endothelial wall.

A further step in the inflammatory response is the increase in vascular permeability, required for leukocyte and solute extravasation (99). This occurs predominantly in the post-capillary venules, where the endothelial layer is rich in vesiculo-vacuolar organelles and caveolae. NO has long been known to modulate microvascular permeability (100), but only in recent years has it become evident that NO derived from NOS3 is required for the increase in permeability at postcapillary venules induced by VEGF and PAF (101–103). The mechanisms for this role in increasing the permeability of the endothelial barrier are currently unclear. In part it appears to be controlled by the canonical NO-cGMP-PKG pathway (104–106). However, S-nitrosylation is emerging as a key regulatory mechanism for the control of the machinery that induces the rearrangements of the cytoskeleton, which ultimately leads to endothelial cell contraction. NOS3 translocation from the plasma membrane to the cytosol, compared with the plasma membrane to the Golgi apparatus (as in vasodilatation) appears to be part of the signaling pathway involved in the regulation of vascular hyperpermeability observed in inflammation (32)

## 7. NO-redox balance in cardiac hypertrophy and remodeling

Disease states that impose an increased load on the heart, such as hypertension, aortic stenosis or myocardial infarction induce cardiac hypertrophy and remodeling. Initially, hypertrophy arises as a compensatory mechanism to the increased load, in order to reduce wall stress. However, when prolonged, it results in cardiac dilation and ultimately in heart failure.

Cardiac hypertrophy is characterized by increased myocyte size, increased protein synthesis and activation of the so called ‘fetal gene programming’. This is the expression of fetal genes, including atrial and brain natriuretic peptides in the ventricles, α*-*skeletal actin, β-myosin heavy chain, and downregulation of SERCA2a and PLN (107,108). Nitric oxide and reactive oxygen species have been linked to cardiac hypertrophy due to their effects on the vasculature, but also as signaling molecules in the heart that affect cardiac remodeling. For instance, NOS1 and NOS3 deficiency lead to cardiac hypertrophy (50,109) and each induces expression of different genes (110), although only NOS3-deficient mice are hypertensive. The NO-cGMP-PKG pathway has been shown to have antihypertrophic effects in the heart (111), due to inhibition of the calcineurin-NFAT pathway, which stimulates hypertrophy. S-nitrosylation may also contribute to the regulation of hypertrophy signaling through inhibition of nuclear factor (NF)-κB (112) and IκB (also known as IKK) (113). This may be of relevance, since it has been shown that NF-κB inhibition induces regression of hypertrophy in a model of spontaneously hypertensive rats, independently of the increased load (114). In this sense, NF-κB appears to behave as a redox-sensitive transcription factor, which is controlled by ROS (115) and NO (113,116,117). This may, therefore, be developed as a therapeutic target in order to reverse remodeling. The NOS isoforms involved in this effect, and how they could be manipulated without disturbing the NO-redox balance in the heart, requires further investigation. It is possible that NOS1- or NOS3-derived NO prevents the establishment of hypertrophy, since NOS1-deficient mice develop hypertrophy in the absence of increased blood pressure (50,109) and in NOS3-deficient mice, restoration of normal blood pressure does not prevent hypertrophy (118). In the brain, NOS1 exerts tonic inhibition of the transcription factor NF-κB (119), where NOS1 exerts tonic inhibition of this transcription factor. In addition, in the intestine, NOS1 tonically suppresses the activation of NF-κB (120). NOS may also act indirectly, through inhibition of ROS that activate NF-κB. Notably, ROS are known to induce hypertrophy through activation of NADPH oxidases (121) and uncoupled NOS3 (111). XO may also be involved in remodeling, since chronic pharmacological inhibition of this molecule with allopurinol has been shown to reverse remodeling in rats and dogs with heart failure (122). In this context, molecular evidence for a direct role of XO is absent, but it is likely to activate the transcriptional pathways used by ROS. The role of NOS2 in hypertrophy remains controversial. Whilst beneficial effects of NOS2 deficiency have been reported (123) another study has found it has no effect on this process (124).

### NOS and myocardial infarction (MI)

MI refers to the cardiomyocyte death that occurs following occlusion of coronary flow. In the post-infarct area, a remodeling process occurs, in which cardiomyocytes are replaced by fibrous tissue. The role of NO in MI has been recently studied using transgenic models in which NOS isoforms have been deleted or overexpressed. NOS1-deficient mice displayed higher mortality after MI (left anterior descending coronary artery occlusion) (125). NOS1^−/−^ mice also showed increased ventricular dilatation and hyporesponsiveness to adrenergic stimulation (125,126). At the molecular level, this was associated with increased O2^•−^ production and reduced NO levels following MI in the NOS1-deficient mice, suggesting that NO/O2^•−^ imbalance contributes to the adverse effects in these animals. Notably, wild type animals upregulate NOS1 expression after MI (126,127), indicating a beneficial effect of NOS1, particularly in the context of remodeling. However, modulation of this enzyme may have unintended adverse effects on contractility. The role of NOS1 post MI is not fully understood, but it appears to be significant. It is well-documented that in animal models of MI, NOS1 is upregulated (127,128,129). This has also been reported in failing human hearts in association with increased NO production and NOS1 translocation from the SR to the sarcolemma. The mechanisms for this upregulation and the molecular pathways that govern this translocation are unknown. In a model of ischemia-reperfusion, NOS1 was shown to be translocated to the sarcolemma, where it inhibited Ca^2+^ influx and thus prevented Ca^2+^ overload as a cardioprotective mechanism (130). Indeed, NOS1 overexpression restricts transsarcolemmal Ca^2+^ influx, but as a consequence, decreases the inotropic reserve of the heart (131).

NOS3 has also been shown to be involved following MI. NOS3-deficient mice exhibit increased mortality and adverse remodeling post-MI (132). Conversely, it has been shown that myocardial NOS3 overexpression ameliorates the effects of MI (133,134). The molecular basis for this remains unknown and the role of NOS2 after MI is less clear.

## 8. Nitric oxide and stem cells in the cardiovascular system

The use of cellular therapy for the treatment of a variety of diseases has revolutionized modern medicine, particularly when the condition involves irreversible loss of cells and tissues. This is particularly relevant in the case of MI and ischemia-induced damage, where the loss of viable cardiac myocytes or vascular endothelium is involved (135). Currently, different approaches are being developed in order to obtain particular cells types as candidates for repairing damaged tissues. The most promising ideas have arisen from the use of embryonic stem cells, as these cells are multipotent and able to differentiate into virtually any cell type. In addition, stem cells derived from the bone marrow have shown promising results.

### Embryonic stem cells: Differentiation into cardiomyocytes, the NOS-cGMP-sGC pathway

Embryonic stem cells are derived from the inner cell mass of the pre-implantation embryo and are able to proliferate for prolonged periods (135). They are also pluripotent and have the capacity to differentiate into multiple cell types including cardiac myocytes, endothelial cells, trophoblast cells and hematopoietic cells. NO has been shown to positively modulate the differentiation of ES cells into cardiomyocytes (136). This has been achieved by using NO donors (SNAP and NONOAtes) and also by transducing the cells with NOS2. Endogenously, ES cells receiving treatment in order to induce cardiac cell differentiation exhibit the presence of the NOS protein and RNA in a time-dependent manner (137,138). Furthermore, NO has been shown to be an intermediate of the differentiating effects of oxytocin and arginine-vasopressin (139,140), possibly through the activity of NOS2.

### Endothelial progenitor cells and vasculogenesis

MI and limb ischemia require the establishment of postnatal neovascularization in order to restore the impaired blood flow. It has been shown that angiogenesis and also vasculogenesis occur under these conditions. During vasculogenesis, endothelial progenitor cells (EPCs) are recruited from the bone marrow to the site of injury, where they differentiate into endothelial cells, giving rise to new blood vessels. During this process, EPCs leave the bone marrow, which is composed of stromal cells, fibroblasts and osteoblasts (141,142).

The mechanism of the mobilization of EPCs includes the production of VEGF within hypoxic tissue, which triggers the production of NO in the stromal cells. Once in the circulation, EPCs are attracted to the site of injury by ischemia-induced upregulation of stromal cell-derived factor-1α.

It is hypothesized that NO production by stromal cells activates matrix metalloproteinases (MMPS). More specifically, NOS3-derived NO may activate MMP-9 by S-nitrosylation. This increase in the activity of MMP-9 results in the cleavage of bound Kit ligand, releasing soluble Kit ligand (also known as stem cells factor). EPC mobilization is deficient in NOS3 knock-out mice (141) and is compromised in conditions where NO bioavailability is deficient, such as diabetes (143), aging (144) and coronary disease (145). Organic nitrates are thus a potential pharmacological tool to restore impaired NO availability, and thereby enhance vasculogenesis, although nitrate tolerance is a limiting factor in this situation.

## 9. Conclusions and future directions

The knowledge of NO signaling has substantially grown since the description of endothelial derived relaxing factor. Notably, the recognition of S-nitrosylation as a relevant signaling process involved in diverse physiological and pathological conditions has opened an avenue for the development of novel therapeutic approaches. In addition, the role of S-nitrosylation signaling in the field of stem cell biology requires extensive investigation.
